# Exploring regulatory network of icariin synthesis in *Herba Epimedii* through integrated omics analysis

**DOI:** 10.3389/fpls.2024.1409601

**Published:** 2024-06-12

**Authors:** Xuedong Zhu, Shiqi Wen, Hameed Gul, Pan Xu, Yang Yang, Ximei Liao, Yunling Ye, Zijian Xu, Xiaofang Zhang, Lin Wu

**Affiliations:** ^1^ Fuling Academy of Southwest University/Southeast Chongqing Academy of Agricultural Sciences, Southwest University, Chongqing, China; ^2^ Integrative Science Center of Germplasm Creation in Western China (CHONGQING) Science City, Southwest University, Chongqing, China; ^3^ College of Agronomy and Biotechnology, Southwest University, Chongqing, China; ^4^ Chongqing Key Laboratory of Biology and Genetic Breeding for Tuber and Root Crops, Southwest University, Chongqing, China; ^5^ Key Laboratory of Germplasm Innovation of Upper Yangtze River, Ministry of Agriculture and Rural Affairs, Chongqing, China

**Keywords:** *Epipremnum sagittatum*, *Epipremnum pubescens*, icariin biosynthesis, multi omics analysis, regulatory network

## Abstract

*Herba Epimedii’s* leaves are highly valued in traditional Chinese medicine for their substantial concentration of flavonoids, which play a crucial role in manifesting the plant’s therapeutic properties. This study investigated the metabolomic, transcriptomic and proteomic profiles of leaves from two *Herba Epimedii* cultivars, *Epipremnum sagittatum* (J) and *Epipremnum pubescens* (R), at three different developmental stages. Metabolite identification and analysis revealed a total of 1,412 and 1,421 metabolites with known structures were found. Flavonoids made up of 33%, including 10 significant accumulated icariin analogues. Transcriptomic analysis unveiled totally 41,644 differentially expressed genes (DEGs) containing five encoded genes participated in icariin biosynthesis pathways. Totally, 9,745 differentially expressed proteins (DEPs) were found, including Cluster-47248.2.p1 (UDP-glucuronosy/UDP-glucosyltransferase), Cluster-30441.2.p1 (O-glucosyltransferase), and Cluster-28344.9.p1 (anthocyanidin 3-O-glucoside 2 “-O-glucosyltransferase-like) through proteomics analysis which are involved to icariin biosynthesis. Protein-protein interaction (PPI) assay exhibited, totally 12 proteins showing a strong relationship of false discovery rate (FDR) <0.05 with these three proteins containing 2 leucine-rich repeat receptor kinase-like protein SRF7, and 5 methyl jasmonate esterase 1. Multi-omics connection networks uncovered 237 DEGs and 72 DEPs exhibited significant associations with the 10 icariin analogues. Overall, our integrated omics approach provides comprehensive insights into the regulatory network underlying icariin synthesis in *Herba Epimedii*, offering valuable resources for further research and development in medicinal plant cultivation and pharmaceutical applications.

## 1 Introduction

Epimedium, a botanical remedy immersed in traditional Chinese medicine and known colloquially as horny goat weed or yin-yang-huo, boasts a rich history spanning over a millennium. Revered for its multifaceted therapeutic properties, Epimedium has found widespread use in managing conditions such as osteoporosis, sexual dysfunction, and cardiovascular ailments. Its popularity in Eastern cultures is underscored by its incorporation into functional foods, dietary supplements, and beverages like tea and wine (Ma et al., 2011; Jiang et al., 2015, 2016). Furthermore, various forms including extracts, tablets, and capsules make it accessible to a diverse range of consumers. Recognized by the Chinese Pharmacopoeia, multiple Epimedium species have been identified, each containing a rich array of bioactive compounds. Prenylflavonoids stand out as pivotal constituents, with over 270 compounds identified across 52 species. Among these, prenylated flavanol glycosides emerge as primary bioactive components, exhibiting diverse pharmacological effects (Jiang et al., 2015). Extensive research has delved into the Phytochemistry and pharmacology of Epimedium, with a focus on elucidating its bioactive constituents. Prenylated flavonoids, notably icariin and epimedins A, B, and C, have been identified as key markers for quality assessment and chemotaxonomy (Zhang et al., 2008). However, while these compounds have been extensively characterized, the underlying biosynthetic governing their production in Epimedium remain incompletely understood.

In recent years, efforts to unravel the biosynthetic mechanisms of Epimedium’s bioactive compounds have gained momentum. Establishing an Expressed Sequence Tag (EST) database for *E. sagittatum* has provided crucial insights into the genes involved in flavonoid biosynthesis (Zeng et al., 2010; Yang et al., 2020). This has led to the isolation of key structural genes implicated in anthocyanin biosynthesis, marking significant progress in understanding Epimedium’s metabolic pathways (Qin et al., 2019, 2022). Exceptional compounds like epimedins A, B, and C, along with icariin, exhibit notable immunomodulatory, anti-osteoporotic, and antitumor properties. icariin, a flavonoid compound predominantly found in Epimedium, icariin is believed to enhance libido and sexual function, potentially by increasing nitric oxide levels to improve blood flow to the genital area (Ishak et al., 2017). Beyond its aphrodisiac properties, icariin has been studied for its potential in promoting bone health, with evidence suggesting it could increase bone density and mitigate bone loss, offering promise for conditions like osteoporosis (Wang et al., 2018). Additionally, icariin exhibits anti-inflammatory and antioxidant properties, which may help reduce inflammation by inhibiting inflammatory cytokines and enzymes, and neutralize harmful free radicals, respectively. Such characteristics position icariin as a potential candidate for managing inflammatory conditions and combating oxidative stress-related diseases like cardiovascular disease and certain cancers (Bi et al., 2022). Furthermore, icariin has shown neuroprotective effects in research, potentially safeguarding nerve cells from damage and degeneration, thus holding implications for neurodegenerative disorders like Alzheimer’s and Parkinson’s disease (Jia et al., 2019). Its anti-aging properties, attributed to its antioxidant and anti-inflammatory actions, are also being explored, suggesting its potential in mitigating age-related skin and tissue deterioration. Moreover, icariin’s ability to promote blood vessel dilation and improve circulation may contribute to cardiovascular health by reducing blood pressure and atherosclerosis risk (Zeng et al., 2022). From these perspectives, the understanding of biosynthesis of these compounds is akin to unlocking the plant’s pharmacological potential. While the phenylpropanoid and flavonoid pathways, elucidated in other medicinal plants, provide a framework, further research is warranted to elucidate Epimedium-specific regulatory mechanisms. Enzymatic steps involved in flavonoid biosynthesis include the catalysis of phenylalanine to cinnamic acid, subsequent conversion to p-coumaric acid, and the synthesis of various flavonoids via condensation reactions. Overall, Epimedium’s therapeutic efficacy stems from its rich repertoire of bioactive compounds, particularly prenylated flavonoids (Shi et al., 2022). Molecular research on the biosynthesis of icariin continues to progress, but some studies identified and characterized a glucosyltransferase gene or a prenyltransferase gene responsible for icariin biosynthesis by ectopic expression of them in tobacco leaves and engineered *Saccharomyces cerevisiae* and *Escherichia coli* (Yang et al., 2020; Wang S, et al., 2021). Understanding the intricate biochemical pathways governing their biosynthesis promises to unlock new avenues for harnessing Epimedium’s therapeutic potential in modern medicine.

The integration of multiple omics technologies has revolutionized plant biology research by providing a holistic view of biological systems. Transcriptomics, metabolomics, and proteomics are key components of this approach, each offering unique insights into gene expression, metabolic pathways, and protein function within plant cells (Hong et al., 2016; Kim et al., 2016; Nejat et al., 2018; Jorrin Novo, 2021; Lu et al., 2021). Transcriptomics focuses on analyzing gene expression patterns at the mRNA level, shedding light on which genes are actively transcribed under specific conditions. This information reveals the dynamics of gene regulation and identifies key pathways involved in plant growth, development, and responses to environmental stimuli. Metabolomics complements transcriptomics by examining the complete set of small molecules or metabolites present in plant cells. Techniques like nuclear magnetic resonance (NMR) spectroscopy and mass spectrometry (MS) reveal metabolic profiles, elucidating biochemical processes underlying plant physiology, metabolism, and stress responses (Kumar, 2016; Deborde et al., 2017; Qin et al., 2018). Proteomics completes the picture by studying the entire complement of proteins expressed within plant systems. By identifying, quantifying, and characterizing proteins, proteomics techniques uncover protein function, post-translational modifications, and interactions, providing valuable insights into plant biology. Two proteins, leucine-rich repeat receptor kinase-like protein SRF7 and methyl jasmonate esterase 1, emerge as central figures in the intricate landscape of plant biology (Song et al., 2022). Despite their distinct functions, these proteins converge in their significant impact on both plant defense mechanisms and physiological processes, serving as crucial orchestrators of responses to environmental cues and stressors (Huan et al., 2022). Integrating data from transcriptomics, metabolomics, and proteomics enables researchers to unravel complex molecular networks and gain a comprehensive understanding of plant biology. Correlation analyses identify genes, proteins, and metabolites that are interconnected, aiding in the reconstruction of metabolic pathways and regulatory networks (Toubiana et al., 2013; Tolani et al., 2021). This integrated approach offers a holistic understanding of gene function and regulation in plants, facilitating discoveries crucial for agriculture, biotechnology, and environmental science. By combining transcriptomics, proteomics, and metabolomics analyses, researchers can identify novel genes, biomarkers, and metabolic pathways with applications in improving crop yield, stress tolerance, and sustainability in agriculture. In the present study, we conducted a multi-omics analysis of Epimedium leaves, focusing on three developmental stages across two species; *E. sagittatum* and *E. pubescens*. Our aim was to construct a comprehensive network elucidating flavonoid biosynthesis, particularly targeting icariin. By comparing the leaves of the two Epimedium species, we were able to identify distinct patterns in icariin and its analogue accumulation. These findings provide valuable reference information for future studies investigating the accumulation of the metabolites in Epimedium. Moreover, our results contribute to the identification of icariin, a flavonoid with heightened medicinal value, paving the way for further exploration of its therapeutic potential and applications in medicine and healthcare.

## 2 Materials and methods

### 2.1 Sample collection

**Figure 1:**
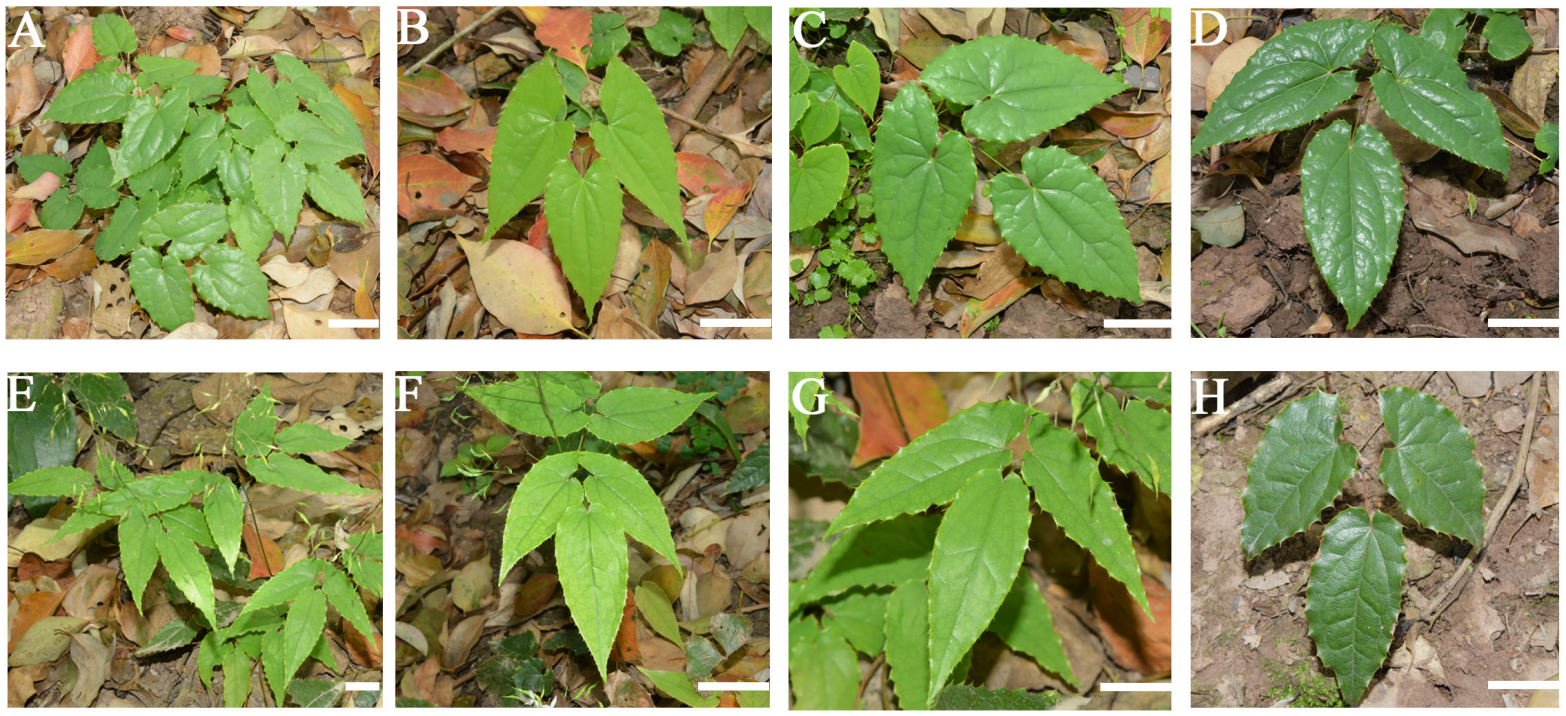
The Herba Epimedii cultivars including *E. sagittatum* (J) and *E. pubescens* (R), at three different developmental stages. **(A–D)** indicating *E. sagittatum* (J) and **(E-H)** indicating *E. pubescens* (R). **(A, E)** shows the whole plant, **(B, F)** fully opened leaves, **(C, G)** slightly leathery leaves and **(D, H)** leathery leaves. The interval between each stage is 7–8 days. Bar =2 cm.

A few researchers employ transcriptome analysis to identify candidate genes associated with the biosynthesis of active ingredients in *E. sagittatum*, including icariin, offering valuable genomic resources for further investigation (Zeng et al., 2010; Yang et al., 2020). In a previous study reported that the contents of icariin analogues in *E. sagittatum* leaves (approximately 7.07%) were significantly higher than that in *E. pubescens* (approximately 4.77%) (Guo and Xiao, 2003). To understand the molecular mechanism resulted in the difference of icariin abundances in those two species, here we used the two cultivars of Epimedium namely *E. sagittatum* (J) and *E. pubescens* (R). Samples of leaves of these two cultivars were collected from Fuling academy of Southwest University/Southeast Chongqing academy of agricultural sciences, China. We collected the samples of leaves at three developmental stages designated as fully opened leaves (stage 1), slightly leathery leaves (stage 2), and leathery leaves (stage 3) (**Figure 1**). After collecting the samples with three biological replicates, we immediately put them into liquid nitrogen and then stored at −80°C for metabolomic, transcriptomic and proteomic analysis.

### 2.2 Sample extraction and metabolome analysis

In the current study, we followed the following protocol for extraction and analysis of metabolites from Epimedium leaves. Firstly, biological samples underwent freeze-drying using a Scientz-100F freeze-drying machine under vacuum conditions, and then grinding into a fine powder using a Retsch MM 400 grinder. Further, the powdered sample was weighted 50 mg by using an electronic balance (MS105D) and added the 1200 µL of pre-cooled 70% methanol-water internal standard extraction solution into each weighted sample. Then vortex the sample mixture at 30s-time intervals every 30 min for total 6 times and subsequently centrifuge the samples at 12000 rpm for 3 min to obtain the supernatant. The supernatant was then filtered through a microporous membrane (0.22 µm pore size) and stored in sampling bottles. Quality control samples (QC) are prepared by mixing sample extracts and used to analyze the repeatability of samples under the same processing method. During the instrument analysis process, one QC is generally inserted into every 10 detection and analysis samples to monitor the repeatability of the analysis process.

UPLC-MS/MS analysis was conducted using an ExionLC™ AD tandem mass spectrometry system coupled with an Agilent SB-C18 chromatographic column. Mobile phases consisted of ultrapure water with 0.1% formic acid (Phase A) and acetonitrile with 0.1% formic acid (Phase B). The elution gradient started with 5% Phase B, linearly increased to 95% over 9 minutes, maintained at 95% for 1 minute, then reduced to 5% over 1.1 minutes, and equilibrated for 3 minutes. Mass spectrometry conditions included an ESI temperature of 550°C and collision-induced ionization with nitrogen gas set to medium. Multiple Reaction Monitoring (MRM) mode was employed for accurate quantification, with a specific set of MRM transitions monitored for each metabolite. Data analysis was conducted using a self-built database (MWDB), and peak areas of chromatographic peaks were integrated and corrected for metabolite quantification.

### 2.3 RNA isolation and transcriptomics

The RNA-seq methodology employed in this study involved meticulous steps from sample collection and preparation to data analysis. Initially, RNA quantification and qualification were conducted using gel electrophoresis, NanoPhotometer^®^ spectrophotometry, and the Bioanalyzer 2100 system to ensure high-quality RNA samples. Following this, library preparation for transcriptome sequencing utilized the NEBNext^®^ UltraTM RNA Library Prep Kit, involving mRNA purification, cDNA synthesis, and adaptor ligation, followed by size selection and PCR amplification. The resulting libraries were then clustered and sequenced on an Illumina platform, generating 150 bp paired-end reads. For data analysis, quality control measures were implemented using fastp to filter out reads with adapters, excessive N content, or low-quality bases. Transcriptome assembly was performed using Trinity, with corset used for transcript clustering. Candidate coding regions within transcript sequences were predicted using TransDecoder. Gene functional annotation was conducted using databases such as Nr, Swiss-Prot, Trembl, Kyoto encyclopedia of genes and genomes (KEGG), gene ontology (GO), EuKaryotic Orthologous Groups/Clusters of Orthologous Groups (KOG/COG), and Pfam. Gene expression levels were quantified using RSEM, and differential expression analysis was performed using DESeq2. Differential gene enrichment analysis was conducted based on the hypergeometric test, with pathway and GO term enrichment analyzed for KEGG and GO databases, respectively (Kanehisa et al., 2021). Transcription factor analysis was carried out using the iTAK for plants (Zheng et al., 2016).

Additionally, weighted correlation network analysis (WGCNA) was performed using the WGCNA R Package (Langfelder and Horvath, 2008). Overall, this comprehensive RNA-seq method provided insights into gene expression, functional annotation, differential expression, transcription factor analysis, protein-protein interaction, and other aspects of transcriptome biology, facilitating a deeper understanding of the molecular mechanisms underlying the studied biological systems.

### 2.4 Integrative metabolomic and transcriptomic and proteomic analysis

Transcriptomic, metabolomic and proteomic data from *Herba Epimedii* leaves were subjected to comprehensive analysis. Specifically, DEGs involved in flavonoid biosynthesis and variations in flavonoid abundance among comparison groups, each comprising three replicates, were meticulously investigated. To elucidate potential correlations between flavonoid biosynthesis genes and transcription factor-encoded genes, Spearman’s correlation coefficients were computed. Genes exhibiting correlation coefficients > 0.80 and correlation *p-values* < 0.05 were selected for further analysis. This integrative approach provides valuable insights into the regulatory networks governing flavonoid metabolism in *Herba Epimedii*, shedding light on potential targets for genetic and metabolic engineering to enhance the medicinal properties of this plant species.

### 2.5 Protein extraction and proteomics

Proteins were extracted from the samples using the acetone precipitation method. The samples were first homogenized using L3 buffer, which contained 1% SDS, 100mM Tris-HCl, 7M urea, 2M thiourea, 1mM PMSF, and 2mM EDTA, after being ground into a fine powder in liquid nitrogen. Centrifugation of the supernatant was used to extract the protein solution after 10 minutes of ultrasonic cracking of ice after vigorous shaking and mixing. The protein solution was then mixed with four times the volume of frozen acetone, and the mixture was allowed to precipitate overnight at a temperature of -20°C. In order to preserve the protein pellet, the precipitate was then centrifuged at 4°C. After being cleaned with cold acetone, the pellet was dissolved in 8M urea. Finally, the protein concentration was determined using a BCA kit following the manufacturer’s instructions.

Tryptic digestion was performed on equal volumes of proteins from each sample using the following protocol: 200 µL of the supernatants were supplemented with 8M urea, reduced to 45 minutes at 37°C with 10 mM DTT, and then alkylated for 15 minutes at room temperature in a dark environment with 50 mM iodoacetamide (IAM). The combination was then treated with four times the volume of cold acetone, and it precipitated for two hours at -20°C. Centrifugation was followed by air drying and resuspension of the protein precipitate in 200 µL of 25 mM ammonium bicarbonate solution and 3 µL of Promega trypsin, which was then digested for an entire night at 37°C. After being digested, the peptides were dried using a vacuum concentration meter, desalted with a C18 Cartridge, concentrated by vacuum centrifugation, and then redissolved in 0.1% (v/v) formic acid. Efficient digestion of proteins into peptides and subsequent preparation for LC-MS/MS analysis were ensured by this process.

Liquid chromatography (LC) was conducted on a nanoElute UHPLC system (Bruker Daltonics, Germany) to separate approximately 200 ng of peptides within 60 minutes. A commercially available reverse-phase C18 column with an integrated CaptiveSpray Emitter (25 cm x 75 μm ID, 1.6 μm, Aurora Series with CSI, IonOpticks, Australia) was used to perform the separation at a flow rate of 0.3 μL/min. An integrated Toaster column oven was used to keep the temperature of the column at 50°C. Formic acid was used to create mobile phases A and B, with 0.1 vol.% in water and 0.1% in ACN, respectively. Over the course of the first 45 minutes, mobile phase B was gradually increased from 2% to 22%, then to 35% over the next 5 minutes, and then to 80% over the next 5 minutes, where it remained for 5 minutes. Using a CaptiveSpray nano-electrospray ion source (CSI), the LC system was connected online to a hybrid timsTOF Pro2 mass spectrometer (Bruker Daltonics, Germany). With ten PASEF MS/MS frames in a single frame, the timsTOF Pro2 ran in the Data-Dependent Parallel Accumulation-Serial Fragmentation (PASEF) mode. Both the MS and MS/MS spectra were obtained between 100 and 1700 m/z, with the capillary voltage set to 1400 V. From 0.7 to 1.4 Vs/cm^2, the ion mobility range (1/K_0) was observedAn intensity threshold of 2500 was used in conjunction with a recurring schedule that had a “target value” of 10,000. As a function of mobility, collision energy was increased linearly from 59 eV at 1/K_0 = 1.6 Vs/cm^2 to 20 eV at 1/K_0 = 0.6 Vs/cm^2. When the m/z was less than 700 and greater than 800, the quadrupole isolation width was set at 2 Th. Quadrupole isolation windows were set by the instrument control software in diaPASEF mode, based on the TIMS scan time. By altering the instrument control circuitry, all applied voltages were ramped synchronously and seamlessly. In particular, a total of 64 windows, or 25 Th isolation windows, were identified from m/z roughly 400 to 1200. The remaining parameters were in line with the DDA-PASEF mode. Comprehensive peptide separation and the capture of excellent spectra for subsequent proteome analysis were made possible by this sturdy LC-MS/MS apparatus.

DIA-NN (v1.8.1) was used to evaluate the MS raw data using a library-free technique. In this investigation, a spectra library was created using deep learning algorithms of neural networks using the MWXS-23–1050-c_20230712.fasta database, which has a total of 86261 sequences. The DIA data was processed with the MBR option to create a spectral library. This library was then used for a new analysis of the data. The false discovery rate (FDR) of search results was reduced to less than 1% at both the protein and precursor ion levels in order to guarantee high confidence in the identification results. Subsequent quantification analysis was conducted using the remaining identifications, yielding dependable insights on the proteome composition of the materials.

### 2.6 qRT-PCR analysis

Six genes were selected randomly for validation using qRT-PCR. RNA extracted from the samples was reverse-transcribed to complementary DNA (cDNA) using PrimeScriptTM RT reagent Kits (TaKaRa, Dalian, China). Subsequently, qRT-PCRs were conducted using the SYBRfiPremix ExTaq TM (TaKaRa, Dalian, China) on a Stepone Real-Time PCR System (ABI, USA). Primer sequences specific for qRT-PCR were designed utilizing Beacon Designer 8.0 and are listed in **Supplementary Table S1**. The thermocycling conditions were as follows: an initial denaturation step at 95°C for 5 min, followed by 40 cycles of denaturation at 95°C for 30 s, annealing at 59°C for 30 s, and extension at 72°C for 30 s. The relative gene expression levels were calculated using the 2−ΔΔCT method, with three biological replicates analyzed for each gene assayed (Livak and Schmittgen, 2001). Primers’ sequences were listed in **Supplementary Table S1**. *Actin* gene was used as housekeeping gene for qRT-PCR (Yang et al., 2020).

### 2.7 Statistical analysis

All samples from the metabolome, transcriptome, and proteome analyses were derived from three independent biological replicates to ensure robust statistical analysis. Statistical analyses were conducted using SPSS 22.0 software (IBM, Chicago, IL, USA). This statistical approach allows for the assessment of significant differences between the two groups, providing insights into the variations observed in metabolite, transcript, or protein abundance between the two cultivars.

## 3 Results

### 3.1 Identification of metabolites and differentially accumulated metabolites

**Figure 2:**
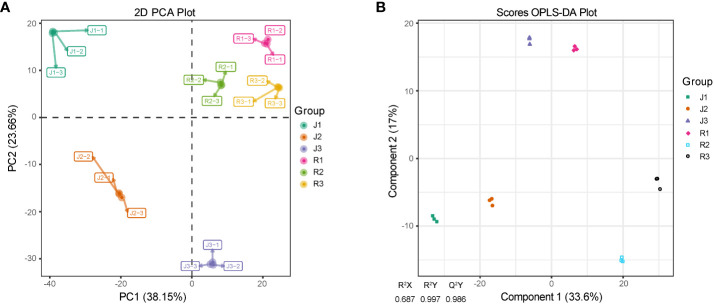
PCA and OPLS-DA scores plots derived from ultra-performance liquid chromatography electrospray ionization-tandem mass spectrometry (UPLC-ESI–MS/MS) profiling of leaves in Herba Epimedii. **(A)** PCA scores plot of the samples of two cultivars; the x-axis represents the PC1 and the y-axis represents PC2. **(B)** OPLS-DA scores plot of the putatively annotated metabolites. The x-axis represents the score value of main components in the orthogonal signal correction process and the differences between the groups can be seen from the direction of the x-axis; the y-axis represents the scores of orthogonal components in the orthogonal signal correction process and the differences within the groups can be seen from the direction of the y-axis. J1-J3/ R1-R3 referred to the three developmental stages of *E*. *sagittatum* (J) and *E*. *pubescens* (R), respectively. .

The metabolomic study of *E. pubescens* (R) and *E. sagittatum* (J) was performed on their leaves. Consistent retention periods and peak intensities were found in total ion chromatography (TIC) (**Supplementary Figure S1**). This suggests high instrument stability, making the results suitable for further analyses. The data obtained from leaves at three different developmental phases were subjected to principal component analysis (PCA), the results showed that principal components 1 (PC1) and 2 (PC2) accounted for 38.15% and 23.66% of the variance, respectively (**Figure 2A**). Orthogonal partial least squares discriminant analysis (OPLS-DA) was used to further investigate the metabolite profiles of *Herba Epimedii*. The results showed a high level of model reliability, with R^2^X, R^2^Y, and Q^2^Y values of 0.687, 0.997, and 0.986, respectively (**Figure 2B**). This demonstrated the OPLS-DA model’s reliability and stability. Indicating diverse metabolic responses influenced by leaf structure and developmental phases in *Herba Epimedii*, score plots from both PCA and OPLS-DA showed clear distinction among the three developmental stages.

**Figure 3:**
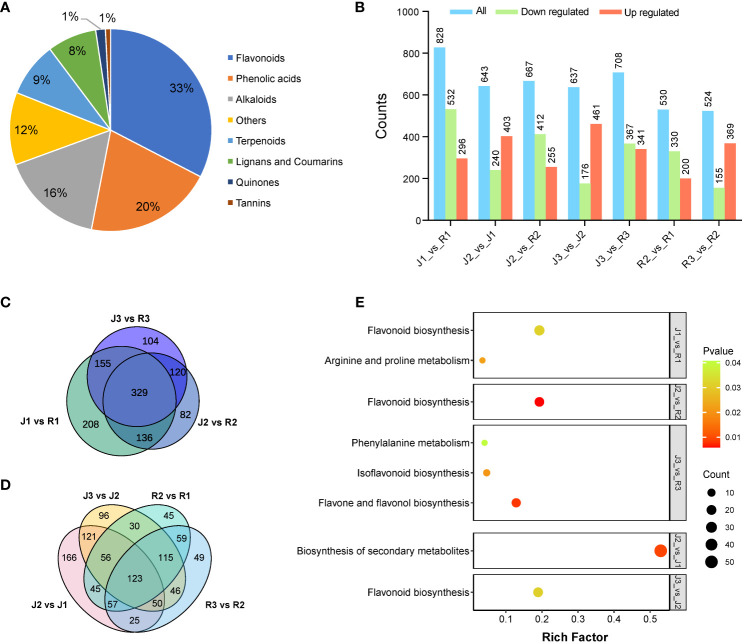
Component analysis of the putatively annotated metabolites and pathway enrichment analysis of the DAMs. **(A)** Component analysis of the putatively an-notated metabolites from leaves. The percentage after each compound represents the percentage of the number of DAMs of a certain class of compounds in the total DAMs. **(B)** Comparison of DAMs among the two cultivars at three different developmental stages. **(C, D)** Venn diagram of DAMs between cultivars and developmental stages, respectively. **(E)** Differential metabolite KEGG enrichment plot.

In the leaves of *E. sagittatum* (J) and *E. pubescens* (R), respectively, a total of 1,412 and 1,421 metabolites with known structures were found; each had three biological replicates for quality validation. **Supplementary Tables S2**, **S3** provide comprehensive details on the discovered metabolites, including compounds, classifications, molecular weights, ionization models, and Kyoto Encyclopedia of Genes and Genomes (KEGG) pathways. Flavonoids made up 33%, phenolic acids 20%, alkaloids 16%, terpenoids 9%, lignans and coumarins 8%, quinones and tannins 1%, and other metabolites made up the remaining 12% of the leaves of *E. sagittatum* (J) and *E. pubescens* (R) (**Figure 3A**).

Differentially accumulated metabolites (DAMs) were identified as those with a variable impact projection (VIP) value of > 1 and a fold change of > 2 or < 0.5. The Kyoto Encyclopedia of Genes and Genomes (KEGG) was used to perform pathway enrichment analysis in order to clarify the biological processes linked with flavonoid DAMs and their functional implications. Fascinatingly, DAMs were significantly enriched in flavonoid pathways (**Figure 3E**), suggesting that leaves have a major impact on flavonoid metabolism in *Herba Epimedii*. DAMs across cultivars at different phases was found through comparative study. In the comparison of J1_vs_R1, 828 DAMs implicated in flavonoid metabolism were found in leaves, of which 532 were downregulated and 296 were upregulated. When comparing *E. sagittatum* (J) to *E. pubescens* (R), flavanol DAMs made up the majority of the elevated metabolites (**Figure 3B**). There were 643 DAMs in J2_vs_J1, 403 of which were upregulated and 240 of which were downregulated; in J2_vs_R2, there were 667 DAMs, 255 of which were upregulated and 412 of which were downregulated. Comparably, 637 DAMs were found in J3_vs_J2, 461 of which were upregulated and 176 of which were downregulated, while 708 DAMs were found in J3_vs_R3, 341 of which were upregulated and 367 of which were downregulated. Furthermore, 530 DAMs were found in R2_vs_R1, 200 of which were upregulated and 330 of which were downregulated, and 524 DAMs were found in R3_vs_R2, 369 of which were upregulated and 155 of which were downregulated (**Figure 3B**). Venn analysis showed a total of 329 DAMs shared significant accumulation comparing J and R. Moreover, 123 DAMs shared significant accumulation comparing three developmental stages (**Figures 3C, D**).

Further, we screened out 10 metabolites related to the icariin synthesis pathway, including Baohuoside I; Icariside II, Epimedin B, Icariside I, Icariin, Delphinidin-3,5-di-O-glucoside), Icaritin, Kaempferol (3,5,7,4’-Tetrahydroxyflavone), Cryptochlorogenic acid (4-O-Caffeoylquinic acid), Delphinidin-3-O-galactoside, and Epimedoside A (**Table 1**). Moreover, through a comparative study across various phases in both cultivars, it was observed that J1_vs_R1 demonstrated superior performance, as illustrated in (**Table 1**).

**Table 1:** Detailed information on Icariin-related metabolites.

Index	Compounds	Class I	Class II	J2_vs_J1_Log2FC	J3_vs_J2_Log2FC	R2_vs_R1_Log2FC	R3_vs_R2_Log2FC	J1_vs_R1_Log2FC	J2_vs_R2_Log2FC	J3_vs_R3_Log2FC
MWSslk054	Baohuoside I; Icariside II	Flavonoids	Flavonols	--	--	--	--	4.55E+00	3.99E+00	3.26E+00
pmp000988	Epimedin B	Flavonoids	Flavonols	--	--	--	--	--	-1.02E+00	--
MWSHY0043	Icariside I	Flavonoids	Flavonols	--	--	--	--	2.17E+00	1.23E+00	--
mws0787	Icariin	Flavonoids	Flavones	--	--	--	--	2.52E+00	3.06E+00	2.72E+00
MWSHY0036	Baohuoside II	Flavonoids	Flavonols	--	--	--	--	2.89E+00	1.13E+00	--
Zblp001600	Delphinidin-3,5-di-O-glucoside	Flavonoids	Anthocyanidins	--	-2.48E+00	3.38E+00	-3.95E+00	4.06E+00	--	--
pmp000973	Icaritin	Flavonoids	Flavonols	-1.30E+00	--	1.74E+00	--	5.16E+00	2.12E+00	2.44E+00
mws1068	Kaempferol (3,5,7,4'-Tetrahydroxyflavone)	Flavonoids	Flavonols	-2.23E+00	--	--	--	1.01E+00	--	-1.63E+00
Zblp001862	Delphinidin-3-O-galactoside	Flavonoids	Anthocyanidins	-1.29E+00	1.25E+00	--	--	--	-2.77E+00	-1.34E+00
pmp000983	Epimedoside A	Flavonoids	Flavonols	-1.41E+00	4.73E+00	--	1.59E+00	--	--	2.73E+00

### 3.2 Transcriptomic profiles of *E. sagittatum* (J) and *E. pubescens* (R) leaves and WGCNA analysis

**Figure 4:**
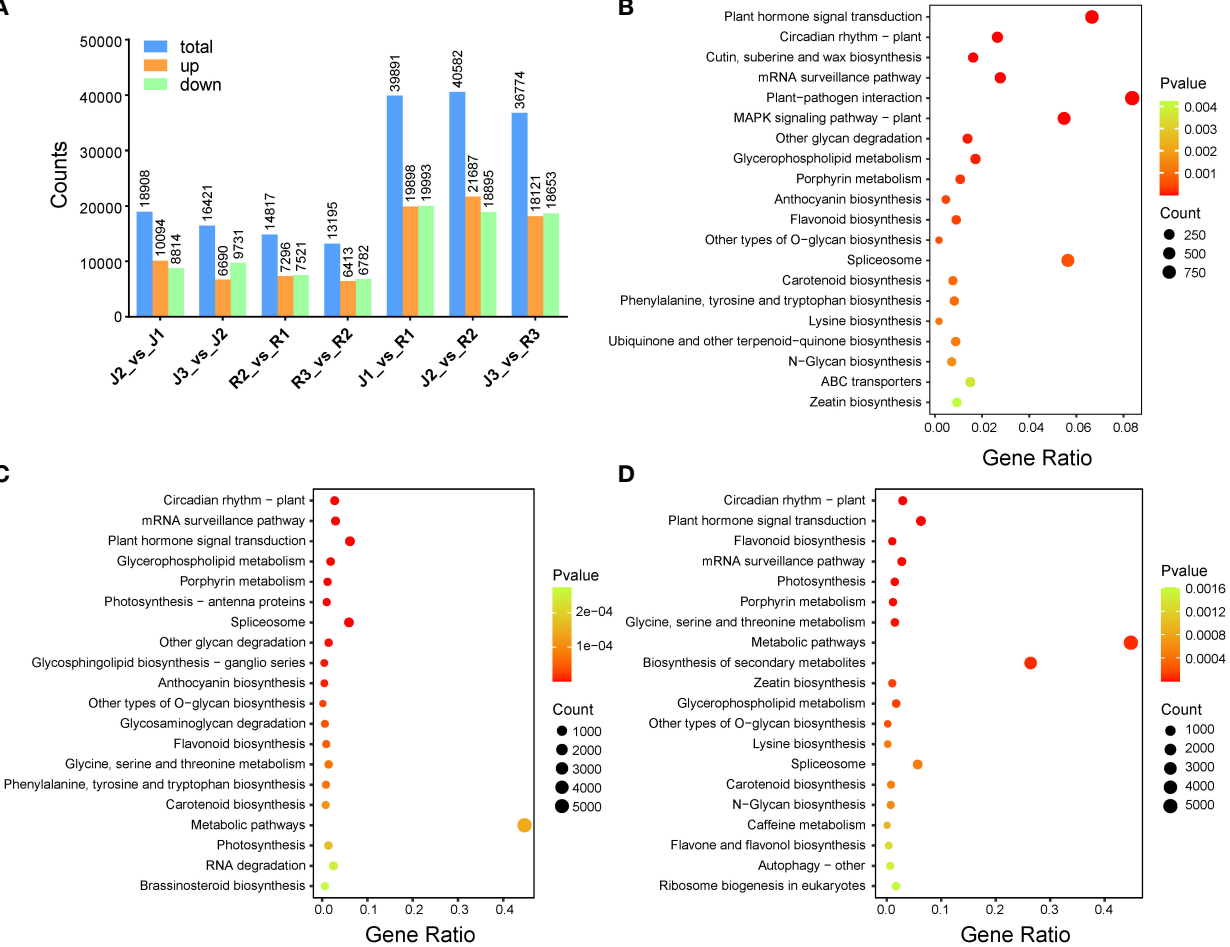
Component analysis of DEGs among the two cultivars and developmental stages of leaf development. **(A)** Comparison of DEGs among the two cultivars at three different developmental stages. **(B-D)** Differential genes GO enrichment plot at three leaf development stages. .

The expression profiles of *E. sagittatum* (J) and *E. pubescens* (R) were scrutinized via RNA-sequencing (RNA-seq) analysis conducted on leaves. Notably, Q20 and Q30 base percentages in (J1-J3) were consistently ≥98.12% and ≥94.42%, respectively, while in (R1-R3), these percentages were ≥98.08% and ≥94.33% (**Supplementary Table S4**). The GC content ranged from 44.84% to 45.55% in (J1-J3) and from 45.48% to 46.01% in (R1-R3). Totally, we found 41,644 differentially expressed genes (DEGs) (**Supplementary Table S5**). Specifically, in the comparison (J2_vs_J1), a total of 18,908 genes exhibited differential expression, with 10,094 upregulated and 8,814 downregulated. Similarly, in (J3_vs_J2), 16,421 genes displayed differential expression, with 6,690 upregulated and 9,731 downregulated (**Figure 4A**). Furthermore, in (R2_vs_R1), 14,817 genes demonstrated differential expression, comprising 7,296 upregulated and 7,521 downregulated genes. Likewise, in (R3_vs_R2), 13,195 genes showed differential expression, with 6,413 upregulated and 6,782 downregulated. Additionally, in (J1_vs_R1), a total of 39,891 genes were differentially expressed, with 19,898 upregulated and 19,993 downregulated. In the comparisons (J2_vs_R2) and (J3_vs_R3), a total of 40,582 and 36,774 genes were differentially expressed, with 21,687 and 18,121 upregulated and 18,895 and 18,653 downregulated, respectively (**Figure 4A**). Consistently, KEGG analysis indicated that DEGs were also significantly enriched in flavonoid pathways (**Figures 4B-D**).

Further, we performed the WGCNA analysis. For this purpose, we were filtering the genes with an average FPKM value of less than or equal to 1 in the transcriptome expression matrix, the gene set for WGCNA analysis was obtained. The soft threshold is set to 18, and a total of 20 modules are obtained after TOM verification (**Supplementary Figures S2A, B**). Additionally, correlation analysis was conducted between 20 modules and 18 samples. The correlation analysis results found that the turquoise module genes (9,397) were specifically expressed in the J cultivar at stage1 while the blue module genes (7,606) were specifically expressed in the R cultivar at stage1. The genes in these two modules may be helpful for research on inter-variety specificity, as shown in (**Supplementary Figures S2C, D**). Furthermore, the magenta and salmon modules are expressed in J2 and R2 samples, the black module is expressed in J1 and R1 samples, the pink module is expressed in J3 and R3 samples. The genes in these four modules may be helpful for studying the developmental stages of Epimedium (**Supplementary Figure S2B**). The expression pattern of the hub genes of these modules were presented in (**Supplementary Table S6**) in all three developmental stages among the cultivars. The above seven modules were selected as candidate modules.

**Figure 5:**
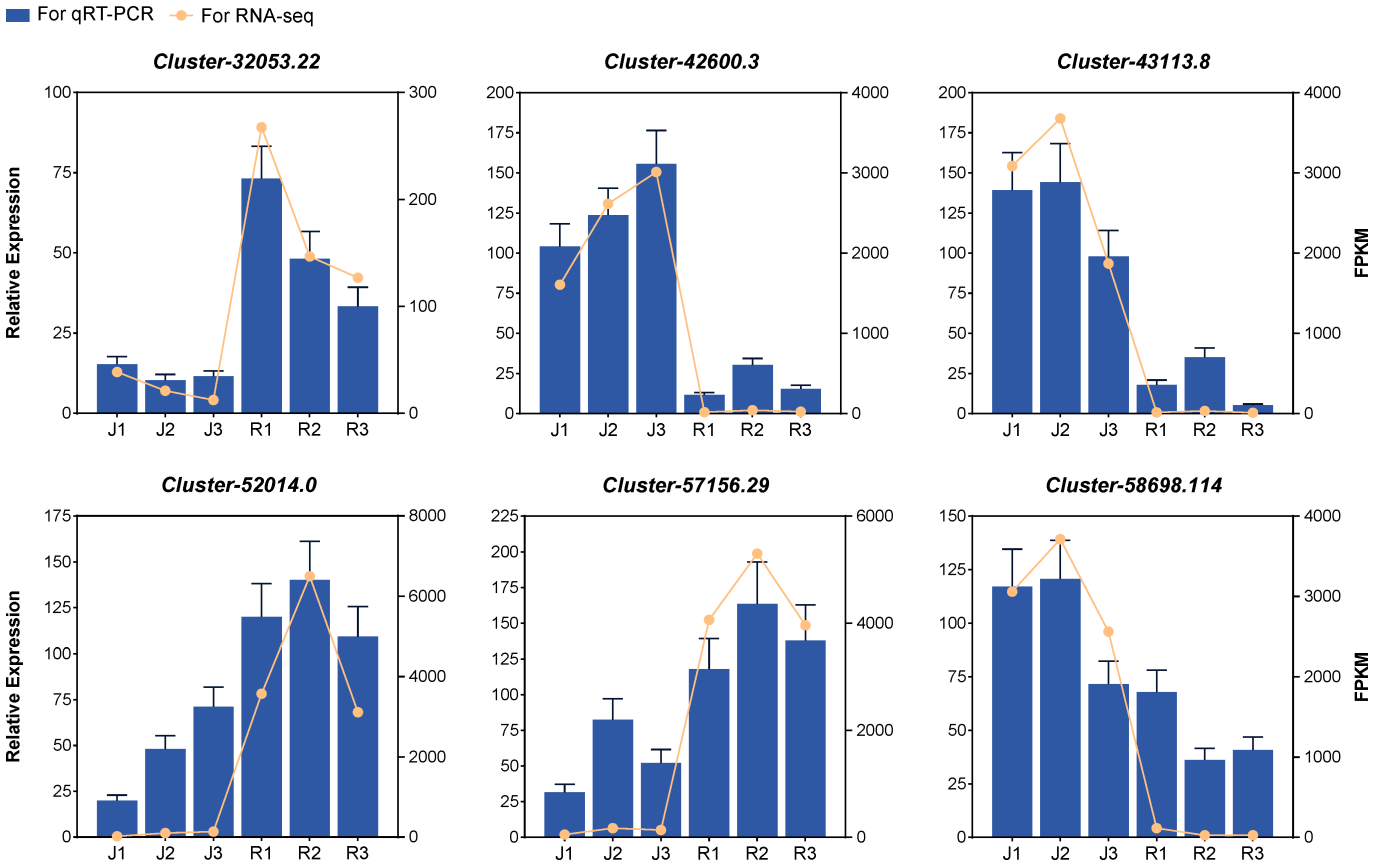
Relative gene expression level and FPKM of random genes in leaves at three developmental stages.

6 genes related to flavonoids and anthocyanin biosynthesis pathways were monitored by qRT-PCR. The expression patterns of the selected DEGs determined by qRT-PCR were consistent with those in the RNA-seq data. This suggested that the results of our RNA-seq data were highly reliable (**Figure 5**).

### 3.3 Proteomic analysis

**Figure 6:**
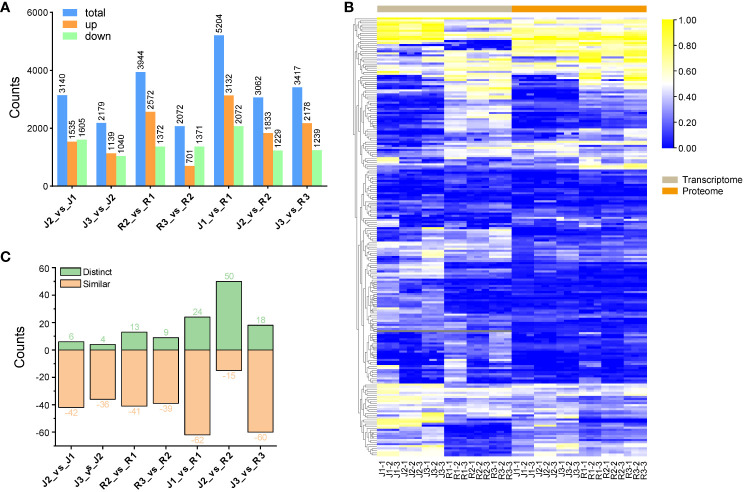
Comparison of DEPs **(A)** Comparison of DEPs among the two cultivars at three different developmental stages. **(B)** Heat map of expression pattern of DEPs associated with DEGs **(C)** Comparison of expression patterns of pathway-related DEGs and DEPs.

Proteins that showed a fold change of 1.5 or less were classified as differentially expressed proteins (DEPs), and a *p-value* of less than 0.05 indicated a significant difference. Using KEGG, pathway enrichment analysis was carried out in order to gain further insight into the role of flavonoid DEPs and the biological processes that are associated with them. Among the cultivars, 9,745 DEPs were found (**Supplementary Table S7**). In specifics, in comparison to (J1_vs_R1), a total of 5,204 DEPs implicated in flavonoid metabolism were found in leaves; of these, 2,072 were downregulated and 3,132 were upregulated. In (J2_vs_J1), A total of 3,140 DEPs from which 1,535 DEPs were upregulated and 1,605 were downregulated while (J2_vs_R2), a total of 3,062 DEPs, 1,833 DEPs were upregulated and 1,229 were downregulated. Of the 2,179 DEPs in (J3_vs_J2), 1,139 showed an upregulation and 1,040 showed a downregulation; of the 3,417 DEPs in (J3_vs_R3), 2,178 showed an upregulation and 1,239 showed a downregulation. In addition, for (R2_vs_R1), a total of 3,944 DEPs showed 2,572 upregulated and 1,372 downregulated DEPs, whereas for (R3_vs_R2), a total of 2,072 DEPs showed 701 upregulated and 1,371 downregulated DEPs (**Figure 6A**). Additionally, we conducted a comparison of the expression patterns between DEPs and their corresponding DEGs. Our analysis revealed that the majority of these transcriptomes and proteomes exhibited similar trends (**Figure 6B**). Following this, we screened pathway-related DEGs and their corresponding DEPs across seven comparison groups. The results unveiled a significant consistency in expression patterns between most pathway-related DEGs and DEPs. However, an intriguing observation emerged from the “J2_vs_R2” comparison group, where the majority of pathway-related DEGs and corresponding DEPs displayed opposite expression patterns (**Figure 6C**).

### 3.4 Association analysis of the icariin related DAMs, DEGs and DEPs

**Figure 7:**
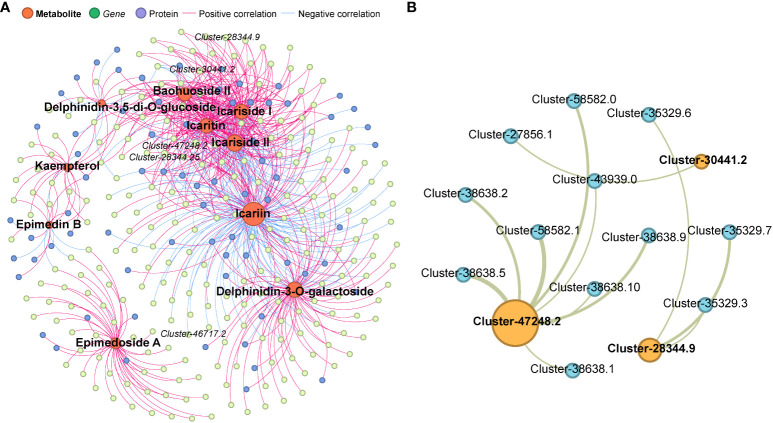
**(A)** Multi-omics connection networks uncovered 237 DEGs and 72 DEPs exhibited significant associations with the 10 Icariin analogues. **(B)** Protein-protein interaction (PPI) assay of 3 Icariin biosynthetic enzyme with 12 proteins showing strong relationship (FDR<0.05) with them. .

After identifying 10 DAMs related to icariin, we conducted further investigation into the regulation of icariin biosynthesis by analyzing the involvement of DEGs and DEPs. Multi-omics connection networks were established using a correlation index (P<0.05). In total, 237 DEGs and 72 DEPs exhibited significant associations with these DAMs (**Figure 7A**, **Supplementary Table S8**). Among the identified DEGs, 32 belonging to anthocyanin-containing compound biosynthetic process (GO:0009718) and 66 belonging to flavonoid metabolic process (GO:0009812) were identified. Additionally, five encoded genes were found to participate in icariin biosynthesis pathways, including Cluster-47248.2 (UDP-glucuronosy/UDP-glucosyltransferase), Cluster-46717.2 (UDP-glucuronosyl/UDP-glucosyltransferase), Cluster-28344.25 (anthocyanidin 3–0-glucoside 2”-0-glucosyltransferase-like), Cluster-30441.2 (0-glucosyltransferase), and Cluster-28344.9 (anthocyanidin 3–0-glucoside 2”-0-glucosyltransferase-like). Notably, three proteins, including Cluster-47248.2.p1 (UDP-glucuronosy/UDP-glucosyltransferase), Cluster-30441.2.p1 (0-glucosyltransferase), and Cluster-28344.9.p1 (anthocyanidin 3–0-glucoside 2”-0-glucosyltransferase-like), displayed significant relationships with these DAMs. To figure out the interaction of three DEPs, protein-protein interaction (PPI) assay was carried out, totally 12 proteins showing strong relationship (FDR<0.05) containing 2 leucine-rich repeat receptor kinase-like protein SRF7, and 5 methyl jasmonate esterase 1 (**Figure 7B**, **Supplementary Table S9**).

## 4 Discussion

*Herba Epimedii*, which is endemic to China, has been utilized in traditional Chinese medicine for millennia. It is valued for its alleged therapeutic benefits, which include its application as an aphrodisiac and for the management of a number of illnesses, including osteoporosis and exhaustion (Wang et al., 2016; Shi et al., 2022). *Herba Epimedii* leaves’ high flavonoid content is one of the main factors that makes them valuable for extraction (Zhang et al., 2008). A broad class of phytonutrients called flavonoids is present in cereals, fruits, vegetables, tea, and wine. They are well-known for having antioxidant qualities and possible health advantages. Critical metabolic genes in medicinal plants have been well studied in the past using transcriptome and metabolic analysis in a variety of organs (Lou et al., 2014; Rai et al., 2017; Adejobi et al., 2021; Chen et al., 2021; Duan et al., 2023). Specifically, a study on A. roxburghii revealed 14 genes involved in the manufacture of flavonoids and 10 genes encoding transcription factors, highlighting the significance of these pathways in medicinal plants (Chen et al., 2020). Further, Chen et al. (2021) revealed the flavonoid metabolic differences between *Anoectochilus roxburghii* and *Anoectochilus formosanus* by integrated analysis of metabolomic and transcriptomic profiles from the leaves. They found the genes as chalcone synthase (CHS), chalcone isomerase (CHI), flavonol synthase (FLS), caffeoyl-CoA O-methyltransferase (CCOAOMT), and anthocyanidin synthase (ANS) as potentially contributing to the downregulation of flavonoid-related metabolites. Nevertheless, little is known about the chemical processes behind the manufacture of flavonoids in *Herba epimedii* and their composition. The comprehensive investigation into the metabolomic, transcriptomic, and proteomic profiles of *Herba Epimedii* cultivars (*E. sagittatum* and *E. pubescens*) leaves offers valuable insights into the complex interplay between metabolic pathways and developmental stages in these plants. The study employed advanced analytical techniques such as metabolomic profiling, RNA-sequencing, and proteomic analysis to unravel the intricate regulatory network underlying the synthesis and regulation of bioactive compounds, particularly flavonoids and icariin.

In order to clarify the biosynthesis pathways of important medicinal ingredients, transcriptome libraries were created from the leaves of two different Epimedium cultivars using high-throughput sequencing technology. Additionally, the flavonoid metabolites in Epimedium leaves were identified using UPLC-MS/MS analysis. After sequencing, we found 41,644 DEGs, through metabolomics 1,276 DAMs were identified, and 9,745 DEPs were obtained through proteomics. Further analysis found that icariin is mainly related to phenylpropanoid biosynthesis (ko00940), flavonoid biosynthesis (ko00941), and flavones and anthocyanin biosynthesis (ko00942) pathways. Combined with the KEGG pathway annotation results, 10 metabolites related to the icariin synthesis pathway were screened out, 72 differentially expressed proteins related to icariin, and a total of 237 DEGs related to the icariin pathway were identified. The foundation for future research into the metabolic pathways in Epimedium is established by this integrative transcriptome, metabolomic and proteomic datasets. Notably, flavonoids are acknowledged as one of Epimedium’s primary medicinal components. KEGG analysis revealed the identification of many flavonoid metabolites that show variable accumulation in distinct Epimedium tissues.

Metabolomic profiling of Epimedium leaves unveiled important metabolites specifically related to the icariin synthesis pathway, including Baohuoside I; Icariside II, Epimedin B, Icariside I, Icariin, Delphinidin-3,5-di-O-glucoside, Icaritin, Kaempferol (3,5,7,4’-Tetrahydroxyflavone), Cryptochlorogenic acid (4-O-Caffeoylquinic acid), Delphinidin-3-O-galactoside, and Epimedoside A. Icariin, a prominent flavonoid, has demonstrated diverse pharmacological activities including antioxidant, anti-inflammatory, and anti-apoptotic effects. These properties have been associated with preventive and therapeutic benefits in various nervous system disorders such as cerebral ischemia, Alzheimer’s disease, Parkinson’s disease, multiple sclerosis, and depression (Wang P, et al., 2021). While the medicinal benefits of *Herba Epimedii* leaves are well-recognized, metabolomic analysis revealed differential accumulation patterns of metabolites involved in flavonoid biosynthesis between the two cultivars. This suggests that appropriate manipulation of different cultivars may lead to enhanced utilization and efficacy of flavonoids (D’amelia et al., 2018). These findings contribute significantly to the evaluation of medicinal plants and enhance our understanding of the applications of different *Herba Epimedii* cultivars.

In the current investigation, we elucidation of basic metabolic pathways of icariin biosynthesis pathways in plants has highlighted the roles of genes such as RT and GT which are pivotal in synthesizing bioactive components in medicinal plants (Yang et al., 2020). In our study, we found totally five GT which showed significant variations between *E. sagittatum* (J) and *E. pubescens* (R) and the expression patterns consistent with the contents of the icariin at different developmental stages. In the regulatory network, 32 DEGs belonging to anthocyanin-containing compound biosynthetic process (GO:0009718) and 66 DEGs belonging to flavonoid metabolic process (GO:0009812) were identified (**Figure 7**, **Supplementary Table S8**).

CHS is a major enzyme in the flavonoid biosynthesis pathway in plants. Its main function is to catalyze the formation of naringenin chalcone by condensation of one 4-coumaroyl-CoA molecule with three malonyl-CoA molecules. The production of different flavonoids, such as flavanols, flavones, flavanones, anthocyanins, and isoflavonoids, begins with this reaction (Imaizumi et al., 2021; Naik et al., 2022). Plants use flavonoids, which are produced by the activity of CHS, for a variety of purposes. These include coloring, defense against diseases and herbivores, attraction of pollinators, and protection against UV radiation. For example, the F3H enzyme catalyzes the conversion of flavanones, such as naringenin, into dihydroflavonols by adding a hydroxyl group at the C-3 position. Dihydroflavonols are the building blocks of several downstream flavonoids, such as flavonols, anthocyanins, and flavan-3-ols (catechins), which is why this step is crucial. On the other hand, the hydroxylation of flavonoids’ B-ring at the 3’ location is catalyzed by the F3’H enzyme. In particular, it changes flavonoids that don’t have a hydroxyl group at the 3’ position—like naringenin and dihydrokaempferol—into the comparable forms that have been 3’-hydroxylated—like eriodictyol and dihydroquercetin. This hydroxylation step is important for generating the structural diversity of flavonoids and contributes to their biological activities and functions in plants (Saito et al., 2013; Liu et al., 2021; Dai et al., 2022). FLS is an enzyme that contributes to the flavonoid biosynthesis pathway. It catalyzes the transformation of dihydroflavonols into flavonols, which include quercetin and kaempferol, respectively, from dihydroflavonols like dihydrokaempferol. Through the oxidation of dihydroflavonols’ C-3 position, an enzyme event occurs that forms the double bond that is unique to flavanols (Winkel, 2006; Saito et al., 2013; Liu et al., 2021). A subclass of flavonoids called flavanols is well-known for its antioxidant qualities and possible medical advantages. GTs are a diverse group of enzymes that catalyze the transfer of sugar moieties from activated donor molecules (such as UDP-glucose, UDP-galactose, or UDP-rhamnose) to acceptor molecules, resulting in the formation of glycosidic bonds. Glycosyltransferases are essential for the modification of flavonoid molecules during flavonoid production because they connect sugar groups to hydroxyl groups on the flavonoid backbone. This late-stage alteration, known as glycosylation, can affect the solubility, stability, and biological activity of flavonoids (Breton et al., 2006; Liang et al., 2015; Ardevol et al., 2016). Plants frequently include glycosylated flavonoids, which add to the wide variety of flavonoid compounds present in the natural world.

In the current research, fascinating observation arose from the comparison group ‘J2_vs_R2’, wherein the majority of differentially expressed genes (DEGs) related to pathways and their corresponding differentially expressed proteins (DEPs) exhibited contrasting expression patterns. The observed values underwent meticulous filtering of mRNA data to ensure high autocorrelation and maximal correlation with protein levels, potentially elucidating their divergence from previous findings (Buccitelli and Selbach, 2020). While numerous studies examining individual mRNA-protein pairs omitted correlation coefficient calculations, they observed instances of proteins and mRNA undergoing contrasting changes, underscoring the common disparity in their expression (Koussounadis et al., 2015). Despite the necessity of mRNAs for protein synthesis, factors such as translation variability, protein degradation, and contextual influences can lead to non-proportional protein levels. Conversely, the presence of a protein indicates the presence of corresponding mRNA during protein synthesis, emphasizing that mRNA levels primarily facilitate potential protein synthesis rather than existing as an endpoint in themselves (Koussounadis et al., 2015; Buccitelli and Selbach, 2020).

Overall, the integration of metabolomic, transcriptomic, and proteomic data offers a holistic understanding of the regulatory network driving metabolic changes in *Herba Epimedii* cultivars. These findings not only enhance our knowledge of plant secondary metabolism but also provide valuable insights for the rational design of breeding strategies aimed at improving the yield and quality of bioactive compounds in medicinal plants.

## 5 Conclusion

The multi-omics approach employed in this study elucidates the intricate regulatory mechanism underlying the biosynthesis and regulation of bioactive compounds, particularly flavonoids and icariin, in *Herba Epimedii* cultivars (*E. sagittatum* and *E. pubescens*). Our findings reveal a diverse array of metabolites present in *Herba Epimedii* leaves, with flavonoids emerging as the most abundant class. Differential accumulation of metabolites across developmental stages and cultivars underscores the complex regulatory networks governing plant metabolism. Transcriptomic analysis further elucidates the transcriptional regulation underlying the observed metabolic changes, with the identification of co-expressed gene modules specific to developmental stages and cultivars. Proteomic analysis complements transcriptomic data by identifying differentially expressed proteins involved in flavonoid metabolism. The integration of proteomic and transcriptomic datasets reveals coordinated regulation at the transcriptional and translational levels, highlighting key genes and proteins implicated in icariin biosynthesis pathways. Overall, multi-omics approach paves the way for further exploration of plant secondary metabolism and holds promise for the development of novel therapeutic agents derived from natural sources.

## Data availability statement

The data will be available upon request. The accession number of RNA-Seq raw data in NCBI is (PRJNA1093226).

## Author contributions

LW: Writing – original draft, Writing – review & editing. XuZ: Writing – original draft, Writing – review & editing. SW: Writing – original draft. HG: Writing – original draft. PX: Writing – review & editing. YY: Writing – review & editing. XL: Writing – original draft. YY: Writing – review & editing. ZX: Writing – original draft. XiZ: Writing – review & editing.
