# Supplementary figures and images for: Exploring regulatory network of icariin synthesis in Herba Epimedii through integrated omics analysis
Source: Front Plant Sci. 2024 Jun 12;15:1409601. doi: 10.3389/fpls.2024.1409601 (PMC11203402; doi:10.3389/fpls.2024.1409601)

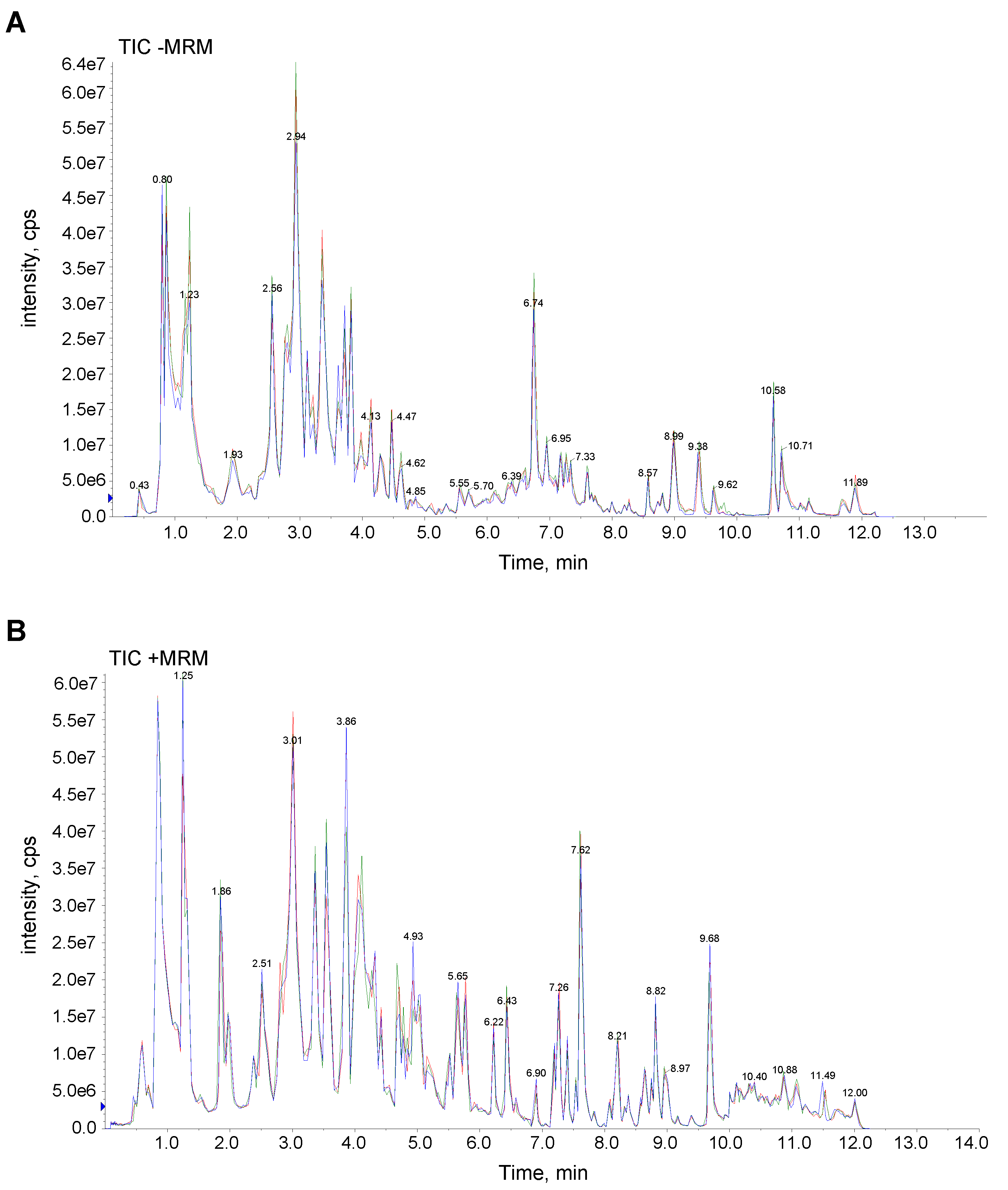

Supplement: Supplementary file 1 [file Image_1.tif]

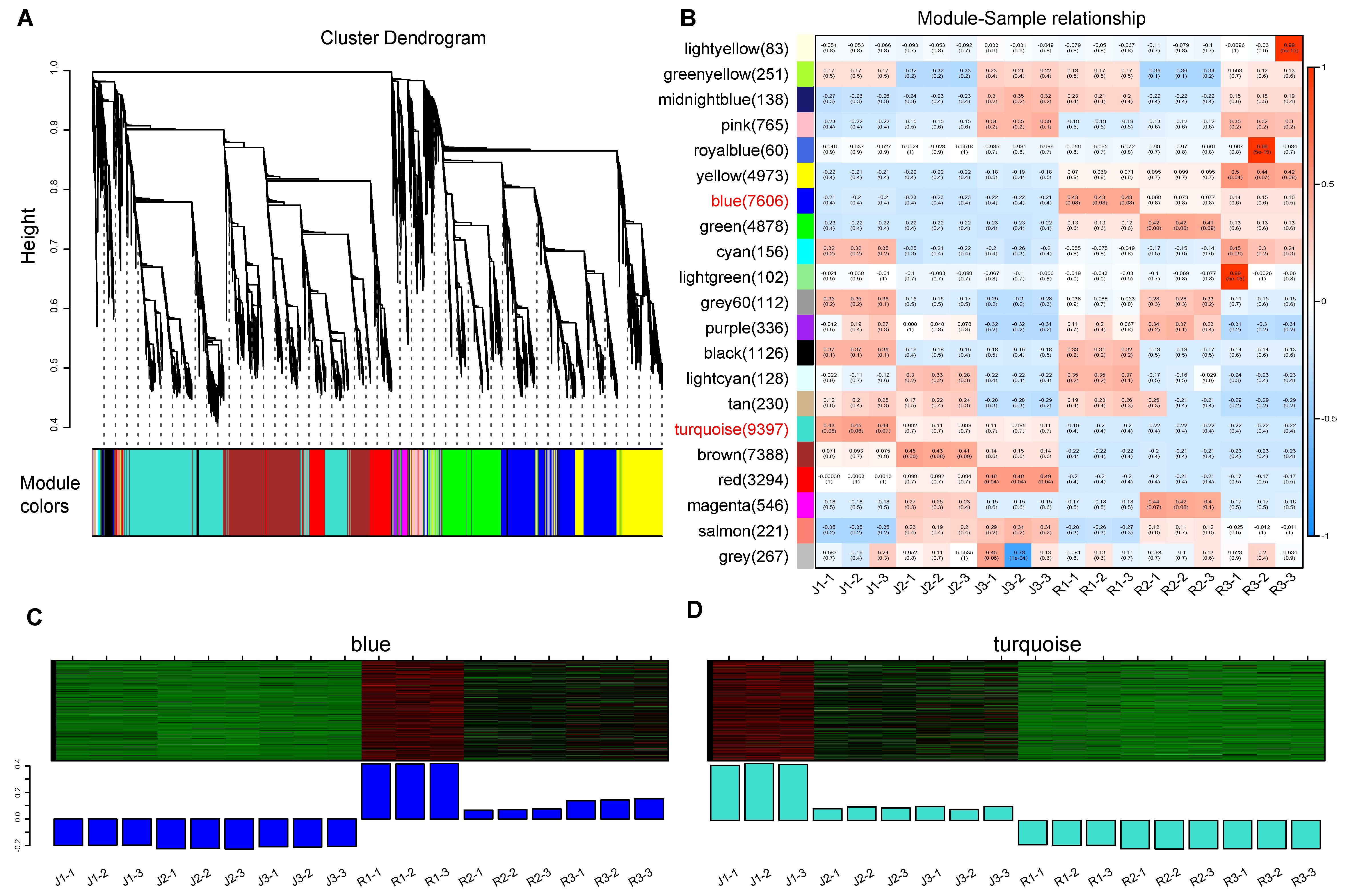

Supplement: Supplementary file 2 [file Image_2.tif]
