# Supplementary material for: Exploring regulatory network of icariin synthesis in Herba Epimedii through integrated omics analysis
Source: Front Plant Sci. 2024 Jun 12;15:1409601. doi: 10.3389/fpls.2024.1409601 (PMC11203402; doi:10.3389/fpls.2024.1409601)
Supplement: Supplementary file 3 [file Table_1.docx]

**Supplementary legends:**

**Fig. S1.** Overlapping analysis of the TIC in QC samples. The x-axis represents the retention time (min) of metabolite detection, and the y-axis represents the intensity of ion current (cps: count per second). (A) for ESI- in mode of leaves. (B) for ESI+ in mode of leaves.

**Fig. S2.** WGCNA module identification and module sample relationships. (A) clustering dendrogram. (B) Heatmap representing the correlation between 20 color modules and two materials, with correlation coeﬃcient and P value < 0.05. (C, D) gene expression patterns in blue module and turquoise module, respectively.

**Table S1.** Primer list.

**Table S2.** Detailed information on the identified metabolites in leaves of *Epimedium sagittatum*(J).

**Table S3.** Detailed information on the identified metabolites in leaves of *Epimedium pubescens*(R).

**Table S4.** Quality control of RNA-seq between *Epimedium sagittatum*(J) and *Epimedium pubescens*(R).

**Table S5.** Matrix FPKM data and annotations for all DEGs.

**Table S6.** Expression information of hub genes.

**Table S7.** Detailed information on all DEPs.

**Table S8.** Detailed information on multi-omics correlation networks.

**Table S9.** Detailed information on protein–protein interaction (PPI) network.
